# Genetic–environment associations explain genetic differentiation and variation between western and eastern North Pacific rhinoceros auklet (*Cerorhinca monocerata*) breeding colonies

**DOI:** 10.1002/ece3.11534

**Published:** 2024-07-11

**Authors:** Brendan A. Graham, J. Mark Hipfner, Kyle W. Wellband, Motohiro Ito, Theresa M. Burg

**Affiliations:** ^1^ Department of Biological Sciences University of Lethbridge Lethbridge Alberta Canada; ^2^ Institute of Arctic Biology University of Alaska Fairbanks Fairbanks Alaska USA; ^3^ Wildlife Research Division Environment and Climate Change Canada Delta British Columbia Canada; ^4^ Fisheries and Oceans Canada West Vancouver British Columbia Canada; ^5^ Faculty of Life Sciences Toyo University Bunkyō‐ku Japan

**Keywords:** adaptive genetic variation, *Cerorhinca monocerata*, local adaptation, neutral genetic variation

## Abstract

Animals are strongly connected to the environments they live in and may become adapted to local environments. Examining genetic–environment associations of key indicator species, like seabirds, provides greater insights into the forces that drive evolution in marine systems. Here we examined a RADseq dataset of 19,213 SNPs for 99 rhinoceros auklets (*Cerorhinca monocerata*) from five western Pacific and 10 eastern Pacific breeding colonies. We used partial redundancy analyses to identify candidate adaptive loci and to quantify the effects of environmental variation on population genetic structure. We identified 262 candidate adaptive loci, which accounted for 3.0% of the observed genetic variation among western Pacific and eastern Pacific breeding colonies. Genetic variation was more strongly associated with pH and maximum current velocity, than maximum sea surface temperature. Genetic–environment associations explain genetic differences between western and eastern Pacific populations; however, genetic variation within the western and eastern Pacific Ocean populations appears to follow a pattern of isolation‐by‐distance. This study represents a first to quantify the relationship between environmental and genetic variation for this widely distributed marine species and provides greater insights into the evolutionary forces that act on marine species.

## Graphical abstract

Examining adaptive genetic variation of key indicator species, like seabirds, provides greater insights into the forces that drive evolution in marine systems. Here we examined a ddRAD dataset of 19,213 SNPs for 99 rhinoceros auklets (*Cerorhinca monocerata*) from five west Pacific and 10 east Pacific breeding colonies. Our analyses provide evidence for local adaption between western and eastern Pacific populations; however, we did not find evidence for local adaptation within populations from either the west Pacific or the east Pacific.

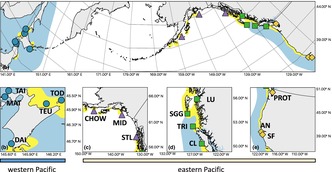

## 1 INTRODUCTION

Examining questions about local adaptation for vagile marine species that have broad distributions is of interest because populations living in different oceanographic regions are exposed to different ecological and environmental conditions. Therefore, ecological adaptation to these environments can lead to reproductive isolation and genetically distinct populations (Friesen, 2015; Friesen et al., 2007; Munro & Burg, 2017). Using genomic analyses, we can build on studies of marine species that used traditional markers (microsatellites and mitochondrial DNA; reviewed in Taylor & Friesen, 2012) to delineate population boundaries and characterize population structure and test for associations between genetic and environmental variation. Examining the relationship between environment and genetic variation for marine species will provide greater insights into the forces generating diversification. Determining the role that environmental variation plays on genetic variation is important (Garcia De Leaniz et al., 2007; Miller et al., 2018; Razgour et al., 2019) given the rapid and substantial climate changes occurring in marine environments (Carozza et al., 2019; Constable et al., 2014; Doney et al., 2012). Studying genetic–environment associations will help to predict how adaptable populations are and how they will respond to changing climates (Capblancq et al., 2020), given that changes in environment can affect fitness (Jenouvrier et al., 2018; Sydeman et al., 2012).

**FIGURE 1:**
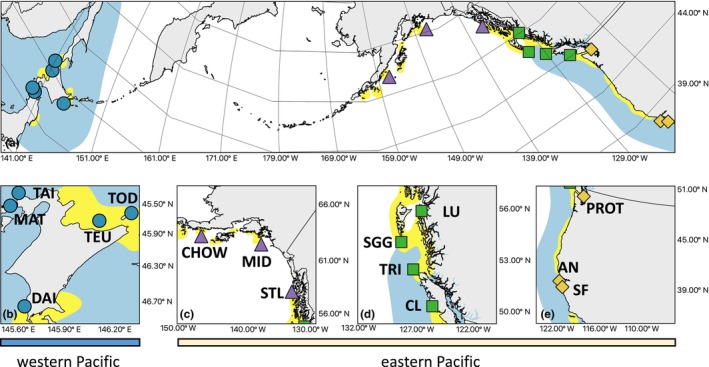
(a) Range map of the rhinoceros auklet (*Cerorhinca monocerata*) across the northern Pacific Ocean. Yellow represents resident areas, while blue represents wintering areas. Shapes represent the 15 breeding colonies where samples were collected from (b) the five breeding colonies sampled from Japan; (c) the three breeding colonies sampled from coastal Alaska; (d) the four colonies sampled from coastal British Columbia; and (e) the three breeding colonies sampled from coastal Washington and coastal California. Breeding colony abbreviations match those found in Table 1. Map was created using QGIS 3.12 with range map layers provided by Bird Life International and Handbokok of the Birds of the World (2022, v 2022.1).

Seabirds offer a compelling system to examine questions about adaptative genetic variation in marine environments because their population dynamics are linked with environmental change and variation (Piatt et al., 2007). For these reasons, seabirds are considered key indicator species of marine systems. Seabirds often exhibit strong natal and breeding philopatry, but philopatry can be quite variable between species (Coulson, 2016). If adult and juvenile philopatry are high, there is potential for local adaptation at relatively small spatial scales (Stiebens et al., 2013) and population genetic patterns may reflect local adaptation. If philopatry is low, however, then the potential for individuals to disperse from their breeding and natal colonies may increase gene flow among colonies (Castillo‐Guerrero et al., 2020; Hipfner et al., 2020; Quillfeldt et al., 2017). As a result, individuals will be exposed to a wider range of environmental conditions and thereby reduce the potential for local adaptation (Antoniou et al., 2021; Jahnke et al., 2022; Lombal et al., 2020). This high dispersal potential is reflected in the complex population genetic patterns found for many species (Burg & Croxall, 2001, 2004; Dhami et al., 2013; González‐Jaramillo & Rocha‐Olivares, 2011; Hall et al., 2009; Milot et al., 2008; Munro & Burg, 2017). In this study, we examine the relationship between genetic and environment variation in the rhinoceros auklet (*Cerorhinca monocerata*), a colonial nesting seabird broadly distributed across the northern Pacific Ocean. This species forages on zooplankton and small schooling fish, which rhinoceros auklets capture underwater during shallow dives (mean dive depth ranges between 9.2 ± 6.7 m and 12.1 ± 5.5 m; Cunningham et al., 2018; Kato et al., 2003). Rhinoceros auklets use both offshore and coastal waters to forage for food depending on the dominant foraging fish community (Iida et al., 2024). Previous studies using microsatellites have shown that populations on either side of the Pacific Ocean are genetically distinct (Abbott et al., 2014; Hipfner et al., 2020). Eastern Pacific Ocean rhinoceros auklets are also smaller than rhinoceros auklets from the western Pacific Ocean (Hipfner et al., 2020). Western and eastern populations forage for different forage fish (Burger et al., 1993; Cunningham et al., 2018; Takahashi et al., 2001; Wilson & Manuwai, 1986) and thereby exhibit foraging behavior and diet differences (Cunningham et al., 2018; Senzaki et al., 2014; Takahashi et al., 2001). Finally, the wintering grounds of eastern and western populations (Figure 1) are also distinct (Hipfner et al., 2020), which may further contribute to the genetic differences previously reported. As oceanic conditions continue to change, in response to climate change, and extreme oceanic events like marine heatwaves (Oliver et al., 2018) become more prominent, questions remain about how climate change will affect marine species like rhinoceros auklets (Wagner et al., 2023). Given that rhinoceros auklets have a broad distribution across the North Pacific, examining how genetic variation is associated with environmental variation in this species can provide greater insight into whether populations are adapted to their conditions and how increasing climate variability will affect populations across their distribution. We used a restriction‐site associated DNA (RADseq) dataset to examine whether genetic differences between western Pacific and eastern Pacific populations are associated with oceanic environmental variation at breeding colonies. Additionally, we examined genetic variation within western Pacific and eastern Pacific populations to determine whether breeding colonies are genetically distinct and whether adaptive genetic variation influences population genetic differences. This last question is especially relevant because rhinoceros auklets breed across a wide geographic area in the northern Pacific Ocean (Figure 1). We hypothesize that sea surface temperature will account for the largest component of genetic variation out of the three environment variables because temperature varies between the eastern and western Pacific Ocean and along a latitudinal gradient within each basin (whereby sea surface temperatures are warmer at southern breeding colonies and cooler at more northern temperatures). Sea surface temperature is a strong predictor of survival and reproduction rates (Jenouvrier et al., 2018; Sydeman et al., 2012) and therefore has a strong effect on seabird fitness. Given that genetic variation and structure in marine systems is associated with population connectivity (i.e., spatial separation during the breeding season; Cowen et al., 2007; Friesen et al., 2007), we evaluate the roles of geographic distance and population connectivity (using least‐cost path data) on genetic variation. Examining multiple hypotheses about the source of genetic variation observed for this widely distributed species will provide greater context and insights into how climate and environmental variation affect genetic variation for marine species.

## 2 METHODS

Between 2010 and 2016, a small blood sample (<1 mL) was collected from 99 adult individuals at 15 distinct breeding colonies (Table 1; Figure 1). Birds were either captured at the burrow, with knockdown or mist nets, at burrow entrances with noose nets, or on the water's surface by hand. As these birds are difficult to sex in the field, we did not record the sex of each bird. Research protocols were approved under the following permits: Western and Northern Animal Care Committee of Environment and Climate Change Canada's Canadian Wildlife Service (15MH01 and 16MH01), US Geological Survey Federal Bird Banding Permit (#09316, #20570), and the Animal Ethics Committee of Hokkaido University, and the Aomori prefecture (#3021).

**TABLE 1:** List of rhinoceros auklet (*Cerorhinca monocerata)* breeding colonies examined in this study, code represents the abbreviation for sampling sites, latitude (Lat), longitude (Long), sample size (*N*), and observed heterozygosity (*H*_o_) and expected heterozygosity (*H*_e_) calculated with the 9349 SNP dataset.

Population	Code	Lat	Long	*N*	*H*_o_	*H*_e_
Western Pacific						
Daikoku	DAI	42.95	144.87	8	0.30	0.28
Taijima	TAI	41.26	140.35	9	0.29	0.28
Matsumae‐Kojima	MAT	41.36	139.82	9	0.29	0.28
Teuri	TEU	44.42	141.31	9	0.29	0.28
Todojima	TOD	45.37	141.04	10	0.29	0.28
Eastern Pacific						
Chowiet	CHOW	56.02	−156.74	4	0.27	0.27
Middleton	MID	59.42	−146.35	3	0.28	0.28
St. Lazaria	STL	56.99	−135.71	6	0.28	0.28
Lucy	LU	54.29	−130.62	6	0.29	0.27
S'Gang Gwaay	SGG	52.10	−131.23	6	0.28	0.27
Triangle	TRI	50.85	−129.07	3	0.24	0.27
Cleland	CL	49.17	−126.09	6	0.26	0.27
Protection	PROT	48.13	−122.93	6	0.29	0.27
Southeast Farallon	SF	37.70	−123.00	5	0.28	0.27
Año Nuevo	AN	37.11	−122.34	9	0.28	0.27

### 2.1 DNA extraction and library preparation

DNA was extracted from blood samples using a salting out extraction protocol (for samples from the eastern Pacific, Miller et al., 1988) or a Qiagen DNeasy kit (for samples from western Pacific). Genomic DNA was used to construct nextRAD genotyping‐by‐sequencing libraries (SNPsaurus, LLC) using the *Sbf1* enzyme as described by Baird et al. (2008). Genomic DNA was first fragmented with Nextera reagent (Illumina, Inc), which also ligates short adapter sequences to the ends of the fragments. The Nextera reaction was scaled for fragmenting 15 ng of genomic DNA, although 20 ng of genomic DNA was used for input to compensate for the amount of degraded DNA in the samples and to increase fragment sizes. Fragmented DNA was then amplified for 27 cycles at an annealing temperature of 74°C, with one of the primers matching the adapter and extending 10 nucleotides into the genomic DNA with the selective sequence GTGTAGAGCC. Only those fragments starting with that sequence can be hybridized by the selective sequence of the primer and efficiently amplified. This protocol resulted in a final library fragment size of 450 bp (Etter et al., 2011). The nextRAD libraries were sequenced on an Illumina NovaSeq 6000 with one lane of single‐end 150 bp reads. All genomic library preparations and sequencing were completed at the University of Oregon.

Sequences were demultiplexed and then trimmed to 122 bp by SNPsaurus using the SNPsaurus pipeline with the bbduk package (BBMap tools, http://sourceforge.net/projects/bbmap/). Next, we assembled reference loci by collecting 10 million high‐quality reads, evenly from all of the samples (~70,000 reads per individual were used), and excluding loci with fewer than seven or more than 700 reads. This range of seven to 700 represents a standardized number calculated by SNPsaurus to retain as many loci as possible without compromising the quality of the data with low‐quality reads. Overall mean depth of the reference genome was 65×. Loci that met the previously stated criteria were then aligned to the assembled reference genome using custom script from SNPsaurus (SNPsaurus, LLC). For the de novo alignment, we mapped 152,204,819 of the original 289,864,865 single‐end reads to the de novo reference genome using an identity threshold of 0.95 using bbmap (BBMap tools). Genotype calling was done using the callvariants tool (BBMap tools), with the following settings (multisample = t rarity = 0.05 minallelefraction = 0.05 usebias = f ow = t nopassdot = f minedistmax = 5 minedist = 5 minavgmapq = 15 minreadmapq = 15 minstrandratio = 0.0 strandedcov = t). The genotype data were converted to a VCF file where we filtered the data to remove loci with a minimum frequency of less than 3%, a *Q*‐score below 20 and removed all individuals with greater than 60% missing data (an additional 13 individuals did not meet this criterion and were omitted from all analyses). The average percentage of missing data was much lower than this original threshold (mean = 5.8% missing data; median = 3.3% missing data), although we included three individuals with 40% missing data because they grouped with other individuals from the same population. To ensure that relatedness did not skew our results, we calculated relatedness among individuals in Genodive 3.04 (Meirmans, 2020). Relatedness among individuals from the same population was <0.08, with exception to one pair that had a relatedness of 0.26, suggesting that one set of full siblings from Teuri were present in our data. Given the low level of relatedness among our data, we retained all samples in our analyses. We retained all of the 19,213 SNPs following the filtering for our examination of genetic–environment associations.

### 2.2 Population genetic structure

To characterize genetic population structure, we filtered our full SNP dataset to allow only a single SNP per contig because SNPs on the same locus are physically linked. SNPs were also checked for linkage disequilibrium in PLINK (version 1.9; Purcell et al., 2007) to further ensure that we did not include any loci that were correlated with each other. To characterize population genetic structure, we calculated Cavalli‐Sforza and Edwards distance (Cavalli‐Sforza & Edwards, 1967) between all individuals and ran a principal coordinate analysis (PCoA) on the distance matrix in PAST 4.11 (Hammer et al., 2001). Additionally, we examined population genetic structure with discriminant analysis of principal components (DAPC) using the adegenet package (Jombart, 2008) in R 4.2.2. The purpose of using this analysis was to provide an alternative method to examine population genetic support to our PCoA. DAPC converts the raw genetic data to principal components and then uses discriminant function analysis to detect population structure. DAPC is considered more sensitive for detecting population structure than PCoA because it reduces within group variation while optimizing between group variation. We ran *k*‐means clustering analysis from 1 to 15 (based on the number of sampled breeding colonies) and selected the optimal *K* by choosing the *K* with the lowest Bayesian information criterion (BIC; Jombart, 2008). Finally, we calculated pairwise *F*_ST_ values between all 15 breeding colonies to characterize the level of genetic differentiation across the range in GENODIVE; deviations from zero were determined using 10,000 permutations.

We downloaded 21 ocean environmental data variables from the Bio‐Oracle database Version 2.2 (https://www.bio‐oracle.org/; Assis et al., 2018). This dataset contains marine data layers for ecologically relevant variables for present day conditions; variables are based on seasonal and monthly averages calculated from the period of 2000–2014. Variables within this dataset have a spatial resolution of 5 arcmin (Assis et al., 2018). Environmental layers were loaded into QGIS 3.28.0 (http://qgis.osgeo.org), and then, we used the point sampling tool to collect environmental data for each breeding colony using the latitude and longitude of each breeding colony as our sampling location. Although rhinoceros auklets breed on islands, the data used represent the marine conditions from the cell that the geographical location falls in. We then tested for intercorrelations between the 21 variables using a Spearman's *r* correlation. Most variables exhibited correlations that exceeded 0.7, which left us with three variables to use in our analysis of genetic and environment associations: (1) maximum current velocity, (2) pH, and (3) maximum sea surface temperature (SST).

We chose these variables because they are biologically relevant to a variety of marine taxa (including whales, urchins, and fish) and are variables that have been analyzed in previous studies as they are thought to influence habitat occupancy, dispersal, and thereby population genetic structure (Antoniou et al., 2021; Banks et al., 2007; Fullard et al., 2000; Lombal et al., 2020). We used these three variables to characterize environmental variation of ocean conditions at breeding colonies because (a) rhinoceros auklets use coastal waters for foraging (Iida et al., 2024); (b) tracking data for foraging trips outside of coastal waters were not available, and (c) we did not have data available for the wintering areas used by the individuals that we collected genetic samples for. Sea surface temperature is likely an important variable for seabirds, given that a review examining the impact of climate change on seabird populations revealed that sea surface temperature negatively affects survival and reproductive success of seabirds in 90% and 70% respectively of all studies reviewed (Sydeman et al., 2012). Less is known about the effect of pH on seabirds, but changes in salinity can lead to increases in energetic costs (Constable et al., 2014; Gutiérrez, 2014) which may further affect fitness. Ocean acidification alters pH and is predicted to reduce coral populations (Hoegh‐Guldberg et al., 2007) which will thereby affect many prey species that depend on the resources provided by these habitats (Chambers et al., 2011; Constable et al., 2014), and has the potential to exhibit large negative effects on the fitness of seabirds, especially if the forage fish they rely on become less abundant and harder to find (Chambers et al., 2011). Finally, oceanic current velocity influences upwelling patterns, which transports nutrients closer to the surface, thereby bringing forage fish closer to the surface. In the absence of upwelling, forage fish remain in deeper waters, forcing individuals to dive deeper for food thereby increasing energetic demands (Soldatini et al., 2023). Although the effect of each environment variable individually affects genetic patterns remains unknown, the combined effect of these factors will affect fitness, a key component of genetic variation and population structure. Additionally, these environmental data affect the prey (including Anchovy [*Engraulus japonicus*], Pacific Sand Lance [*Ammodytus hexapterus*], and Capelin [*Mallotus vilosus*]) that this species relies on, which will also affect seabird fitness. For example, Pacific Sand Lance are less abundant and grow slower under warmer oceanic conditions (Robards et al., 2002), and embryo survival decreases as pH increases (Murray et al., 2019). Given that maximum sea temperature and pH peak during the summer, the variables used in this study reflect environment conditions during the breeding season of rhinoceros auklets.

### 2.3 Partial redundancy analysis

We used redundancy analysis (RDA) to examine genotype–environment associations for rhinoceros auklets. Redundancy analysis combines ordination and regression techniques to analyze the relationship between multiple predictor variables and a single response variable (Legendre & Legendre, 2012). RDA methods are increasingly being used in landscape genomic studies because this analysis provides a flexible and robust method to analyze the relationship between genetic and environmental variation (Capblancq & Forester, 2021; Forester et al., 2018). Redundancy analysis also allows for variance partitioning to examine the contributions of each variable on the response variable. Another advantage of RDA is that partial RDA models allow for covariates to be included to account for any variables that may influence the relationship between genetic and environmental variation (Forester et al., 2018). For our RDA and partial RDA models, we used individual‐based allele count data (genotypes were scored as 0/1/2) for each locus as our full SNP (19,213 SNPs) genetic dataset. For all missing data, we used an imputation approach where we converted missing genotypes to the most common genotype at each locus across all individuals following the approach of Cayuela et al. (2022). Our environmental data consisted of the three variables described above: maximum current velocity, pH, and maximum sea surface temperature. Finally, we included population genetic structure as a covariate in our model to account for the signal of population genetic structure on genotype–environment associations using the first two principal coordinates from our population genetic structure analysis with the 9349 SNP dataset for this variable (Forester et al., 2018). We analyzed alternative hypotheses alongside environment. Given that spatial data can have a strong effect on genetic patterns, we analyzed the effect of isolation‐by‐resistance between the western and eastern Pacific Ocean populations and isolation‐by‐distance within the eastern and western Pacific Ocean populations. We analyzed the effect of isolation‐by‐resistance between the eastern and western populations, as opposed to isolation‐by‐distance, because these two areas are disjunct and separated by deep ocean waters (Hipfner et al., 2020). For this analysis, we used least‐cost corridor resistance surface distance data presented by Prill (2019). We converted these data to continuous variables using a principal component analysis in Past 4.11 and used the first and second principal components as predictor variables in our redundancy models. Within the eastern and western Pacific Ocean, we examined isolation‐by‐distance because Prill (2019) showed that there are no biogeographic barriers preventing individuals from moving between breeding colonies within either the eastern or western Pacific Ocean basins. Therefore, we included latitude and longitude as predictor variables to quantify the effect of geographic distance on genetic patterns. We identified candidate loci using the loading scores for each SNP from the RDA following the approach outlined in Forester et al. (2018). SNPs with loading scores that were ±3 SD (two‐tailed *p*‐value = .003) from the mean score of each of the constrained loading scores were considered outlier SNPs. We used the first three constrained axes as a greater proportion of the variation was explained by these three axes.

To complement our partial RDA, we used the R package latent factor mixed models (LFMM; Caye et al., 2019; Frichot et al., 2013) to identify putative adaptive loci. These models detect correlations between allele frequencies and environmental variation while estimating random effects (including population structure, population history and isolation‐by‐distance) to reduce the number of false‐positive putative loci under selection detected. For these analyses, we used *K* = 2 based on the overall pattern of population genetic structure observed with PCA and used the same three environmental variables, maximum current velocity, pH, and maximum sea surface temperature, that we used for our partial RDA models. Once we ran LFMM, we examined the genomic inflation factor (GIF) to assess how well the models accounted for potential confounding factors in the dataset. We then used a false discovery rate of 0.01 to identify candidate loci below this threshold following the approach of François et al. (2016).

## 3 RESULTS

We generated 279,483,253 high‐quality reads; from these 158,027,685 high‐quality reads from 101,465 contigs were mapped to the reference genome with a mean sequencing depth of 47× and a median sequencing depth of 35×. Our filtering and assembly protocol resulted in a 19,213 SNP dataset for our analyses of the influence of adaptive genetic variation in rhinoceros auklets. After filtering one SNP per locus and accounting for linkage disequilibrium, we were left with 9349 SNPs from the original 19,213 SNP dataset to analyze population genetic structure.

**FIGURE 2:**
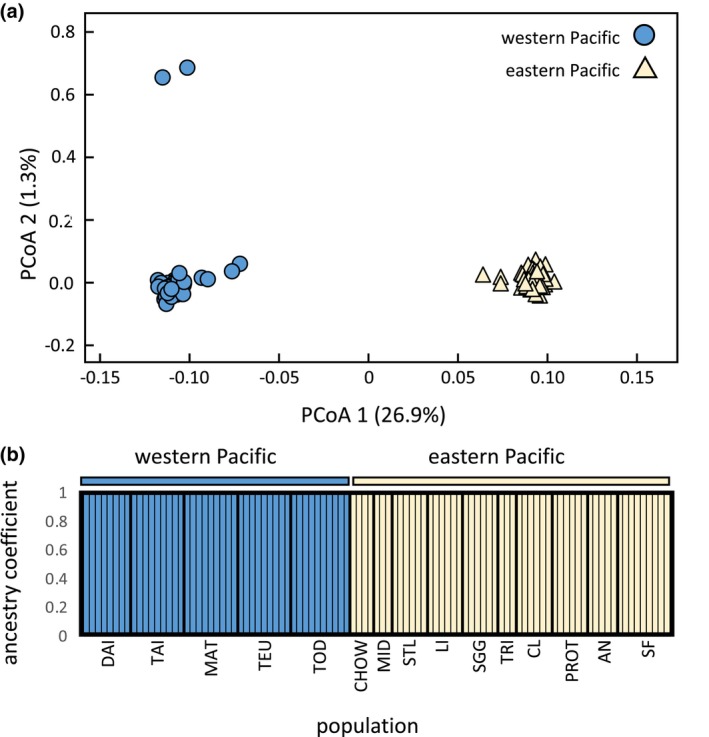
(a) Principal coordinate analysis of 99 rhinoceros auklets (*Cerorhinca monocerata*) using 9349 SNPs. Circles (blue) designate individuals sampled from western Pacific breeding colonies, while triangles (light yellow) designate individuals sampled from eastern Pacific breeding colonies. (b) Shows individual ancestry coefficients for the 99 rhinoceros auklets as determined using DAPC at *K* = 2.

Our analysis of population genetic structure with 9349 SNPs found that western Pacific and eastern Pacific populations of rhinoceros auklets form two distinct genetic clusters (Figure 2a). The first two principal coordinates accounted for 26.9% and 1.3% of the observed variation, respectively. Additionally, DAPC revealed two distinct genetic clusters, separating western and eastern Pacific Ocean populations from each other (*K* = 2_BIC_ = 703.9). The genetic patterns observed in this study match the patterns found by Abbott et al. (2014) and Hipfner et al. (2020) with microsatellite data. Breeding colonies did not form distinct genetic clusters within the western Pacific or eastern Pacific. Western Pacific and eastern Pacific populations are genetically distinct from each other based on pairwise *F*_ST_ values (mean = 0.100 ± 0.001; Table 2), but genetic differentiation among breeding colonies within the western Pacific and eastern Pacific was relatively low; only three pairwise *F*_ST_ comparisons were significantly different from zero among western Pacific Ocean populations, while only three pairwise *F*_ST_ comparisons were significant among eastern Pacific Ocean populations.

**TABLE 2:** Pairwise *F*_ST_ comparisons among populations.

	DAI	TAI	MAT	TEU	TOD	CHOW	MID	STL	LI	SGG	TRI	CL	PROTt	SF	AN
DAI	—	0.768	0.345	0.068	0.314	**0.002**	**0.006**	**<0.001**	**<0.001**	**<0.001**	**0.005**	**<0.001**	**<0.001**	**0.001**	**<0.001**
TAI	0.000	—	0.119	0.029	0.454	**0.003**	**0.005**	**<0.001**	**<0.001**	**<0.001**	**0.004**	**<0.001**	**0.001**	**0.001**	**<0.001**
MAT	0.000	0.001	—	**0.003**	**<0.001**	**0.001**	**0.005**	**<0.001**	**<0.001**	**<0.001**	**0.004**	**<0.001**	**<0.001**	**0.001**	**<0.001**
TEU	0.006	0.006	**0.007**	—	**0.002**	**0.001**	**0.005**	**<0.001**	**<0.001**	**<0.001**	**0.005**	**<0.001**	**0.000**	**0.001**	**<0.001**
TOD	0.001	0.000	**0.004**	**0.007**	—	**0.001**	**0.004**	**<0.001**	**<0.001**	**<0.001**	**0.003**	**<0.001**	**0.001**	**0.000**	**<0.001**
CHOW	**0.104**	**0.102**	**0.101**	**0.105**	**0.101**	—	0.287	0.053	0.170	0.418	0.086	0.154	0.086	0.025	0.034
MID	**0.101**	**0.100**	**0.101**	**0.107**	**0.101**	0.003	—	0.524	0.134	0.049	0.400	0.464	0.235	0.129	0.318
STL	**0.100**	**0.100**	**0.100**	**0.104**	**0.10**	0.003	0.000	—	0.092	0.107	0.085	0.029	0.023	0.283	0.064
LI	**0.099**	**0.097**	**0.098**	**0.101**	**0.098**	0.002	0.003	0.002	—	0.503	0.061	0.393	0.180	0.574	0.405
SGG	**0.097**	**0.097**	**0.096**	**0.099**	**0.096**	0.001	0.005	0.002	0.000	—	**0.050**	0.219	0.553	0.363	0.803
TRI	**0.107**	**0.110**	**0.110**	**0.112**	**0.109**	0.007	0.001	0.008	0.009	**0.010**	—	0.203	0.062	0.129	0.047
CL	**0.103**	**0.103**	**0.104**	**0.105**	**0.102**	0.004	0.000	0.004	0.001	0.002	0.006	—	**0.011**	0.056	0.024
PROT	**0.103**	**0.102**	**0.102**	**0.104**	**0.100**	0.003	0.002	0.004	0.002	0.000	0.011	**0.005**	—	**0.006**	0.248
SF	**0.102**	**0.101**	**0.100**	**0.105**	**0.100**	0.004	0.004	0.001	0.000	0.001	0.006	0.004	**0.005**	—	0.064
AN	**0.105**	**0.104**	**0.102**	**0.106**	**0.103**	0.003	0.000	0.002	0.000	0.000	0.008	0.004	0.001	0.002	—

*Note*: Values below diagonal represent pairwise *F*_ST_ values made among populations with the 9349 SNP dataset. Values above diagonal represent *p*‐values; pairwise *F*_ST_ values were considered significantly different from zero at *p* < .011 indicated in bold, following sequential Bonferroni corrections. Colony names are coded and full colony names can be found in Table 1. Western Pacific Ocean breeding colonies are highlighted in blue, while eastern Pacific Ocean breeding colonies are highlighted in light yellow.

**FIGURE 3:**
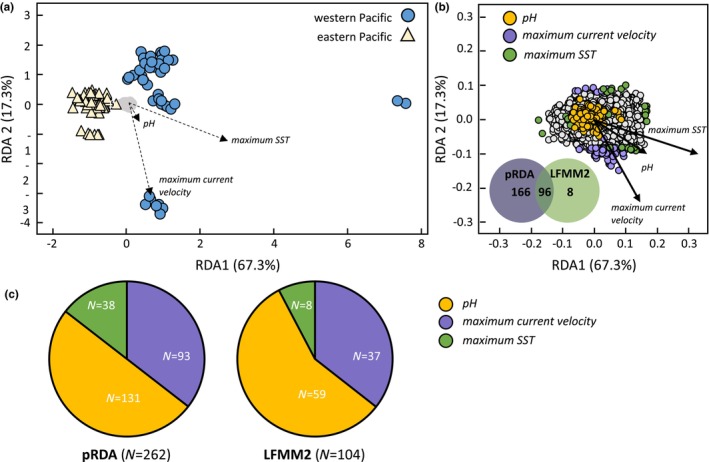
(a) Partial redundancy analysis plot for RDA axes 1 and 2 using 19,213 SNPs incorporating the effect of population genetic structure on adaptive genetic variation. Circles (blue) designate individuals sampled from western Pacific breeding colonies, while triangles (light yellow) designate individuals sampled from eastern Pacific breeding colonies. Arrows represent the direction and magnitude of effect by the three environmental variables (maximum current velocity, pH, and maximum sea surface temperature) examined, and loci are shown in gray. (b) Shows the distribution of loci in the ordination space. Candidate loci associated with specific environmental variables are designated by color: purple = maximum current velocity, yellow = pH, and green = maximum sea surface temperature. Venn diagram compares the number of candidate loci detected in the partial RDA model and LFMM. (c) Pie charts show the number of candidate loci that were associated with the three environment variables for the partial RDA model and LFMM.

Our partial RDA model that used allele count data from 19,213 SNPs and accounted for neutral population genetic structure revealed environmental variation influences genetic variation among rhinoceros auklet populations from the North Pacific. The three environmental variables (maximum sea surface temperature, maximum current velocity, and pH) accounted for 3% of the observed genetic variation (adjusted *r* ^2^ [$r_{\mathrm{adj}}^{2}$] = 3.0%, *p* = .001; Table 3; Figure 3a) when population genetic structure was accounted for. This result indicates that population genetic structure alone does not explain the genetic variation and that genetic–environment associations are present. The three RDA axes explained 67.3%, 17.3%, and 15.4% of the observed variation. Overall, the partial RDA model identified 262 candidate loci that are associated with environmental variation between western and eastern Pacific populations. A greater proportion of loci were associated with pH and maximum current velocity, while fewer loci were associated with maximum sea surface temperature (Figure 3b,c). By comparison, the redundancy models examining the influence of population genetic structure ($r_{\mathrm{adj}}^{2}$ = 4.0%, *p* = .001) and isolation‐by‐resistance ($r_{\mathrm{adj}}^{2}$ = 7.3%, *p* = .001) on genetic variation accounted for a greater proportion of variance than the partial model that examined the influence of environment.

**TABLE 3:** Summary table for the redundancy and partial redundancy models examining the effect of environment, population genetic structure, isolation‐by‐resistance, and isolation‐by‐distance on genetic variation for rhinoceros auklets.

Model	*F*	*p*	df	Inertia	*r* ^2^ (%)	$r_{\mathrm{adj}}^{2}$ (%)
East and West						
Environment | population genetic structure	**2.05**	**.001**	3, 94	1115.0	5.8	3.0
Population genetic structure	5.09	**.001**	1, 97	953.0	5.0	4.0
Isolation‐by‐resistance	8.69	**.001**	1, 97	1572.0	8.2	7.3
East						
Environment | population genetic structure	1.01	.240	3, 49	971.0	5.7	0.1
Population genetic structure	1.04	**.007**	1, 52	331.5	2.0	0.1
Isolation‐by‐distance	1.01	.110	2, 51	648.1	3.8	0.1
West						
Environment | population genetic structure	1.04	**.006**	3, 40	1232.2	7.0	0.3
Population genetic structure	1.48	**.001**	1, 43	585.3	3.3	1.1
Isolation‐by‐distance	1.04	**.050**	2, 42	828.4	4.7	0.2

*Note*: Pseudo‐*F*‐score (*F*), *p*‐value (*p*), degrees freedom (df), inertia, percent variation explained by each variable (*r* ^2^), and adjusted percent variation explained by each variable ($r_{\mathrm{adj}}^{2}$) are reported for each model. All bolded values are models considered significant at *p* < .05.

Latent factor mixed models (LFMM) identified 104 candidate loci (*p* < .01). Ninety‐six loci (Figure 3b) were identified as candidate adaptive loci by both LFMM and partial RDA model. LFMM identified eight additional candidate adaptive loci that were not identified by the partial RDA model, and more loci were associated with pH (*n* = 59) and maximum current velocity (*n* = 37) than with maximum sea surface temperature (*n* = 8; Figure 3c).

**FIGURE 4:**
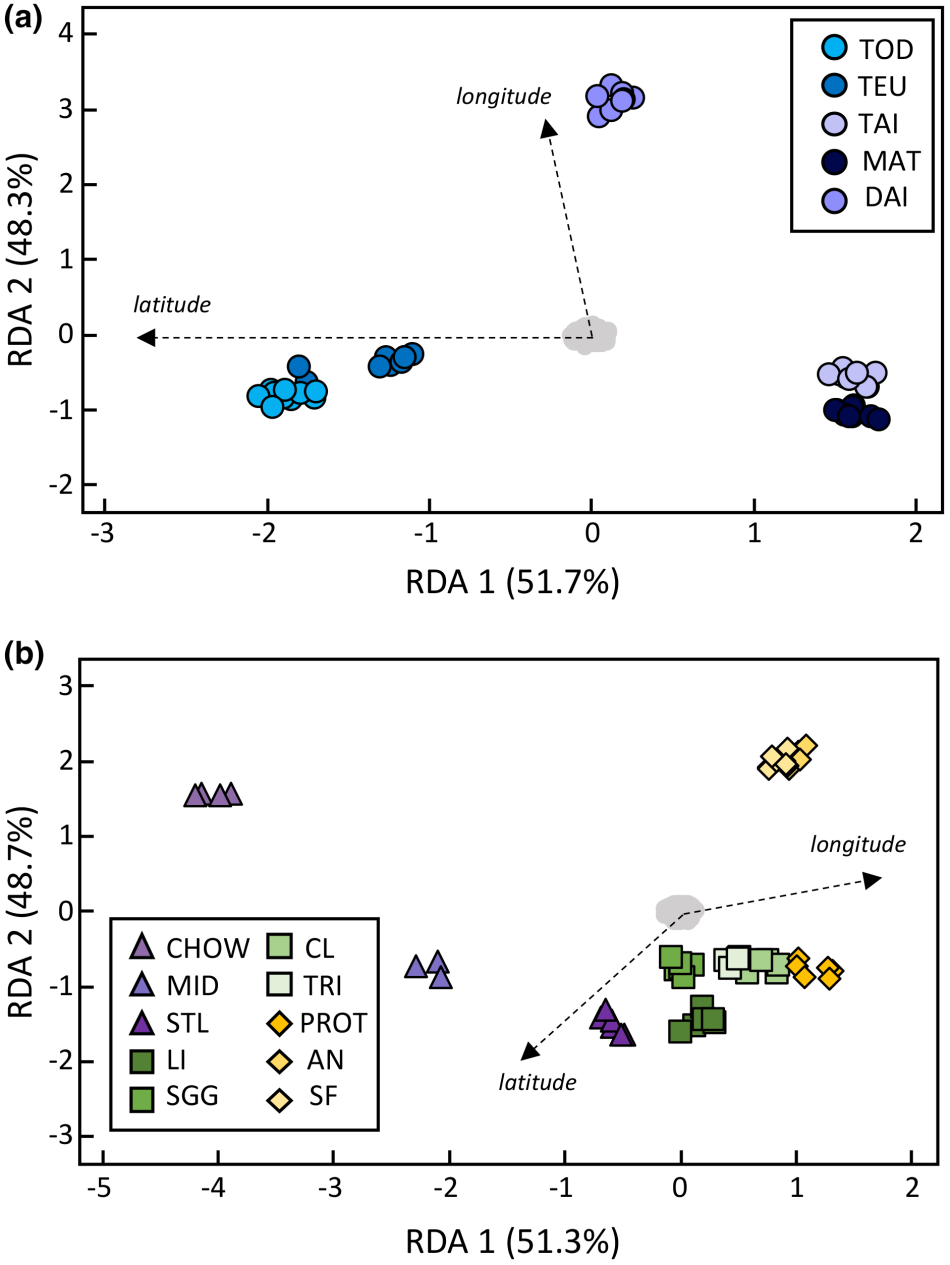
(a) RDA plots for RDA axes 1 and 2 using 19,213 SNPs examining the effect of geographic distance on genetic variation in the western Pacific Ocean. (b) RDA plots for RDA axes 1 and 2 using 19,213 SNPs examining the effect of geographic distance on genetic variation in the eastern Pacific Ocean. Arrows represent the direction and magnitude of the effect by latitude and longitude in the model.

Our model examining genotype–environment associations in the western Pacific accounted for a relatively small portion of the variance ($r_{\mathrm{adj}}^{2}$ = 0.3%, *p* = .006). Redundancy models examining population genetic structure and isolation were also significant and accounted for a relatively low proportion of variance (0.1% and 0.2%, respectively). Genetic variation in the western Pacific appears to be associated with spatial data, given that colonies that are geographically closer appear to cluster together (Figure 4a). The partial redundancy model identified 165 candidate loci, for which the candidate loci and environment associations were evenly distributed across the three environmental variables (maximum current velocity, *N* = 54; pH, *N* = 54; and maximum sea surface temperature, *N* = 57).

Our partial redundancy and redundancy models examining genotype–environment associations in the eastern Pacific were not significant (*p* = .24); environmental variables accounted for a relatively low portion of the observed variance ($r_{\mathrm{adj}}^{2}$ = 0.1%) among eastern Pacific breeding colonies. The model examining population genetic structure was significant but accounted for a low percentage of variation (*r* ^*2*^ = 0.1%, *p* = .007). Isolation‐by‐distance accounted for a slightly higher proportion of variance (*r* ^*2*^ = 0.2%, *p* = .11), and similar to genetic patterns in the western Pacific Ocean, genetic patterns appear to vary with geographic distance (Figure 4b). The colonies further north, Chowiet and Middleton Islands, clustered further away from the other breeding colonies, while the southernmost colonies, Año Nuevo and southeast Farallon, also clustered separately from all other colonies.

## 4 DISCUSSION

Our genetic analyses of rhinoceros auklet breeding colonies across their range provide evidence for genotype–environment associations. Environmental variation accounted for a relatively low but significant amount of the genetic variation observed between western Pacific and eastern Pacific populations; genetic variation was more strongly associated with pH and maximum current velocity than with maximum sea surface temperature based on partial RDA and LFMM analyses. When we compared our three models (environment, population structure (with 9349 SNPs), and isolation‐by‐resistance), the isolation‐by‐resistance model had the highest adjusted‐*r* ^2^ value, which indicates that physical barriers limit gene flow across the range and have a strong influence on genetic variation for vagile species like seabirds (Lombal et al., 2020). That all three of the models examined are associated with genetic variation reflects that multiple factors drive patterns of genetic variation and differentiation. Overall, we found little evidence of genetic–environment associations within each ocean basin where population genetic patterns appear to follow a pattern of isolation‐by‐distance.

Many seabird populations show reduced population genetic structure over large areas because there are relatively few barriers to gene flow in marine systems (Cowen et al., 2007; Friesen et al., 2007; Jahnke & Jonsson, 2022). The limited gene flow based on population genetic structure between the western and eastern Pacific rhinoceros auklet populations observed in this study (as reflected by the PCoA, DAPC, and pairwise *F*_ST_ comparisons with the 9349 SNP dataset and the RDA model examining isolation‐by‐resistance with the full dataset) and previous studies (Abbott et al., 2014; Hipfner et al., 2020) suggest that deep ocean waters in the Bering Sea act as a barrier to dispersal between these populations (Hipfner et al., 2020; Prill, 2019). Prill (2019) noted that rhinoceros auklets appear to disperse readily between colonies within both the eastern and western Pacific Ocean and therefore hypothesized that other ecological or behavioral factors may act to limit dispersal and gene flow between eastern and western Pacific Ocean populations. The pattern of the western Pacific populations being distinct from the eastern Pacific populations has been observed in some vagile seabird species (González‐Jaramillo & Rocha‐Olivares, 2011; Herman et al., 2022), but not all (Pshenichnikova et al., 2015). Population genetic differentiation between the western and eastern Pacific may also reflect the lasting effects of Pleistocene glaciations, where the ranges of the eastern and western Pacific populations shifted southwards isolating them from each other during the Last Glacial Maximum (Abbott et al., 2014).

We detected low levels of genetic differentiation among colonies both within the western and Pacific. Philopatry (Coulson, 2016; Friesen, 2015) and spatial segregation during the non‐breeding season (Friesen et al., 2007; Munro & Burg, 2017) are both thought to drive genetic differentiation in seabirds and likely play a role in local genetic patterns within the western and eastern Pacific for rhinoceros auklets. Like other alcids, rhinoceros auklets are known to exhibit strong breeding philopatry (Hipfner et al., 2020; Morrison et al., 2011), and that genetic patterns appear to follow patterns of isolation‐by‐distance within both ocean basins, suggests that breeding philopatry may explain genetic differences. Non‐breeding season distributions also influence genetic patterns. rhinoceros auklets winter at‐sea outside of the breeding season, and Hipfner et al. (2020) found that genetic differentiation was associated with the extent of spatial overlap during the non‐breeding season; populations that exhibited lower spatial overlap were more genetically differentiated. One other potential factor that could reduce genetic differentiation among breeding colonies within the eastern and western Pacific is the possibility of sex‐linked loci in our dataset. Since we did not have information on the sex of each bird, we were unable to detect and remove putative sex‐linked loci from our dataset.

Adaptive candidate loci were associated with the three environmental variables we examined, but a greater proportion were associated with maximum current velocity and pH. Ocean currents and pH play a key role in the distribution and diversity of prey species in marine systems (Ballance et al., 2006; Bost et al., 2009; Chambers et al., 2011; Munro & Burg, 2017; Young et al., 2015). Therefore, the strong association of pH and maximum current velocity with genetic variation could reflect in the differences in foraging behavior and preferences that exist between eastern and western Pacific Ocean populations (Burger et al., 1993; Cunningham et al., 2018; Senzaki et al., 2014; Takahashi et al., 2001; Wilson & Manuwai, 1986). It is well established that when foraging behavior differs between populations, the formation of ecotypes can occur and these ecotypes may become genetically distinct following reduced gene flow (Abeyrama, 2023; Breistein et al., 2022; de Bruyn et al., 2013; Fruet et al., 2017).

Temperature is associated with energy expenditures for many marine species, including rhinoceros auklets (Shimabukuro et al., 2023), yet we found relatively few adaptive candidate loci associated with maximum sea surface temperatures despite western Pacific populations occurring in noticeably warmer waters than eastern Pacific populations. Temperature has been shown to be an important determinant of genetic variation for other marine species (Antoniou et al., 2021; Bradbury et al., 2010; Limborg et al., 2012; Reusch, 2014) and is thought to act as a key driver of diversification for marine species (Bowen et al., 2016), including seabirds (Torres et al., 2021). It is possible that the relationship between temperature and genetic variation may be more important in exothermic organisms that cannot thermoregulate or behaviorally thermoregulate as opposed to endothermic organism like seabirds. The limited influence of temperature on genetic variation detected in this study may also reflect adaptability by rhinoceros auklets. rhinoceros auklets occupy large home ranges (570,000–2,200,000 km^2^; Hipfner et al., 2020) and inhabit large ranges during the full annual cycle. One of our initial goals was to evaluate this relationship between genetic and sea surface temperature variation, given that sea surface temperatures continue to rise. It is possible that our analysis of this relationship was not sensitive enough (i.e., we did not analyze the whole genome) or other temperature‐related variables may be more important, and therefore, further analyses are required to characterize this relationship. Additionally, our analyses focused exclusively on breeding colony environmental variables. Previous work on Black‐browed Albatross (*Thalassarche melanophris*), a highly vagile seabird, has suggested that environmental variability on wintering grounds exerts greater effects on fitness than environmental variability during the breeding season (Jenouvrier et al., 2018). Based on the results of this previous study, it is possible that breeding colony environmental variation is a poor predictor of genetic variation for rhinoceros auklets. Further examining the environmental variation that rhinoceros auklets encounter on their breeding grounds does not reflect the true scale of environmental variation that individuals encounter because individuals may potentially spend less time on their breeding grounds than on their wintering grounds. Therefore, selection pressures for adaption outside of the breeding season may exert a strong influence on genetic structure and fitness for rhinoceros auklets as suggested by Jenouvrier et al. (2018).

This study quantifies the relationship between environmental variation and genetic variation for rhinoceros auklets. Our analyses suggest that environmental variation explained a low portion of genetic variation between western and eastern Pacific populations. One possible reason for a small influence of environmental variation on genetic variation is the sequencing method used. Although RADseq offers a robust method to examine genetic variation, the power of this sequencing method is noticeably less than whole‐genome sequencing (Szarmach et al., 2021). A comparison of the methods revealed that most high‐*F*_ST_ regions identified with whole‐genome sequencing were not detected with RADseq due to lower genome coverage (Szarmach et al., 2021). The authors noted that whole‐genome sequencing exhibited greater resolution at finer spatial scales when characterizing divergence.

The patterns observed in this study highlight that multiple factors influence genetic differentiation in marine species and that diverse evolutionary forces drive diversification in marine environments. Our results suggest that environmental variation plays a role on genetic variation for this species, but other factors including physical barriers appear to have also had a strong influence on genetic differentiation. Further genetic patterns may be influenced by historical extinction and recolonization by non‐adjacent populations (as in the case of Año Nuevo and Southeast Farallon but see Hipfner et al., 2020), and further studies should examine demographic processes more closely when examining range wide genetic patterns. Our choice to use the full dataset identified 262 putatively adaptive SNPs. It is possible that this decision may have over inflated the number of putatively adaptive loci. Future studies incorporating whole‐genome sequencing methods will likely provide a more accurate account of the number and percentage of putatively adaptive SNPs. Overall, our study provides a first step in studying genetic and environmental associations in this species, that future studies can build on for studying the role of environmental selection on genetic variation in rhinoceros auklets and other seabird species.

## AUTHOR CONTRIBUTIONS

**Brendan A. Graham:** Conceptualization (lead); data curation (lead); formal analysis (lead); investigation (lead); methodology (lead); writing – original draft (lead); writing – review and editing (lead). **J. Mark Hipfner:** Conceptualization (lead); funding acquisition (lead); investigation (equal); methodology (equal); project administration (lead); resources (lead); supervision (equal); writing – review and editing (equal). **Kyle W. Wellband:** Conceptualization (equal); formal analysis (equal); investigation (equal); methodology (equal); writing – review and editing (equal). **Motohiro Ito:** Data curation (equal); funding acquisition (equal); investigation (equal); project administration (equal); writing – review and editing (equal). **Theresa M. Burg:** Conceptualization (lead); data curation (lead); funding acquisition (lead); methodology (equal); project administration (lead); resources (lead); supervision (lead); writing – review and editing (lead).

## CONFLICT OF INTEREST STATEMENT

The authors report no conflict of interests with this manuscript.

## Data Availability

Data will be made available in Dryad following publication at the following Dryad link https://doi.org/10.5061/dryad.ffbg79d39.
